# A Novel Nomogram Based on Log Odds of Metastatic Lymph Nodes to Predict Overall Survival in Patients With Perihilar Cholangiocarcinoma After Surgery

**DOI:** 10.3389/fonc.2021.649699

**Published:** 2021-07-22

**Authors:** Wenbo Zou, Chunyu Zhu, Zizheng Wang, Xianglong Tan, Chenggang Li, Zhiming Zhao, Minggen Hu, Rong Liu

**Affiliations:** ^1^ Medical School of Chinese People’s Liberation Army (PLA), Beijing, China; ^2^ Faculty of Hepato-Pancreato-Biliary Surgery, Chinese PLA General Hospital, Beijing, China; ^3^ Institute of Hepatobiliary Surgery of Chinese PLA, Key Laboratory of Digital Hepatobiliary Surgery, PLA, Beijing, China

**Keywords:** nomogram, perihilar cholangiocarcinoma, overall survival, log odds of metastatic lymph nodes, SEER

## Abstract

**Background:**

Various lymph node staging strategies were reported to be significantly correlated with perihilar cholangiocarcinoma(pCCA) prognosis. This study aimed to evaluate their predictive abilities and construct an optimal model predicting overall survival (OS).

**Methods:**

Patients with pCCA were collected as the training cohort from the Surveillance, Epidemiology, and End Results (SEER) database. Four models were constructed, involving four LNs staging strategies. The optimal model for predicting OS was evaluated by calculation of the concordance index (C-index) and Akaike information criterion (AIC), and validated by using the area under curve (AUC) and calibration curves. The clinical benefits of nomogram were evaluated by decision curve analysis (DCA). A Chinese cohort was collected to be an external validation cohort.

**Results:**

There were 319 patients and 109 patients in the SEER database and Chinese cohort respectively. We developed an optimal model involving age, T stage, tumor size, LODDS, which showed better predictive accuracy than others. The C-index of the nomogram was 0.695, the time-dependent AUC exceeded 0.7 within 36 months which was significantly higher than that of the American Joint Committee on Cancer (AJCC) stage. The calibration curves for survival probability showed the nomogram prediction had good uniformity of the practical survival. The DCA curves exhibited our nomogram with higher clinical utility compared with the AJCC stage and single LOODS.

**Conclusions:**

LODDS is a strong independent prognostic factor, and the nomogram has a great ability to predict OS, which helps assist clinicians to conduct personalized clinical practice.

## Introduction

Cholangiocarcinoma (CCA) is rare biliary tract malignancies with higher aggressive and poor prognosis (1), and pCCA is the most common subtype and makes up to sixty percent of CCA (2). Primary lesion resection and lymph node dissection are routine strategies for pCCA patients until now, but the long-term survival of patients is still not ideal (3). Therefore, increasingly prognostic factors have been identified, such as race, age, tumor size and so on (4, 5), one of these which affected the OS and curative effect after surgery is lymph node metastasis (6, 7). The number of intraoperative lymph node dissection and the status of lymph nodes (LNs) is significantly helpful for evaluating lymph node metastasis (8).

In the 7^th^ AJCC stage for pCCA, the identification of the N stage mainly depends on the metastatic area of LNs (9). According to subsequently numerous clinical studies, the new 8^th^ AJCC stage adopts the number of positive LNs to better predict prognosis (10). However, recent studies indicated it had similar predictive capability compared with the 7^th^ AJCC stage (11, 12). Consequently, it is a little difficult to apply the separate number of metastatic LNs for evaluating the prognosis and need to identify some novel prognostic indicators. Recently, the LNR and LODDS, two novel staging systems for predicting lymph node metastasis, have been convinced by some professors and have shown predictive accuracy for the OS compared with the traditional N stage (13, 14), LNR is equal to the ratio of positive LNs to the retrieved LNs. The LODDS is defined as log [(0.5+ (positive LNs)/(0.5+ (negative LNs))], and they have shown excellent advantages for evaluating prognosis in various malignant tumors and LODDS was deemed as the most predictively accurate method compared with others (15–17). However, only a few articles studied the advantages of LODDS in pCCA patients, and it had not been incorporated into an excellently prognostic model for pCCA patients. Nomogram has been increasingly popular and has the important ability to predict cancer-related patient’s OS individually (18, 19). According to the nomogram score, clinicians could better implement individualized treatments. Furthermore, because of the better accuracy of LODDS, it would become a significant reference for the new staging systems.

In our study, we used cox regression analysis to screen out independent factors and constructed four models involving 7^th^, 8^th^ N stage, LNR, and LODDS. An optimal model predicting OS was selected by the minimal C-index and maximal AIC, and LODDS could become the optimally predictive factors compared with others in the training cohort. Subsequently, a nomogram was established based on the above model, and verified by an external cohort from China, with the aim of popularization and application.

## Methods

### Study Population

Original data was collected from Surveillance, Epidemiology, and End Results (SEER) database of American Cancer Institute (http://seer.cancer.gov/) by SEER*Stat software (version 8.3.8). The inclusion criteria: According to the CS Schema v0204+, and all the patients were diagnosed as pCCA; the exclusion criteria: the patients lacked race, TNM stage, grade classification, tumor size, surgical, and LNs information. Due to the original SEER cohort adopting the 7^th^ TNM stage, after analyzing the 7^th^ and 8^th^ of AJCC stage, only the N stage was altered by using the number of regionally positive LNs. Therefore, we transformed the 7^th^ N stage to the 8^th^ N stage for subsequent analysis. An additional validation cohort including pCCA patients undergoing surgery were collected from the Faculty of Hepato-Pancreato-Biliary Surgery in Chinese PLA General Hospital. The patients were follow-up by telephone or outpatient clinic interview. The study was approved by the ethics committee of the Chinese PLA General Hospital.

### Statistical Analysis

Categorical variables were reported as counts and proportions and analyzed by the chi-squared test for comparison among groups. We used univariate cox analysis to select potentially prognostic factors, and which were further included in multivariate cox analysis to obtain the independent prognostic factors. X-tile software (version 3.6.1) was used to access the optimal cut-off values of variables. According to the optimal cut-off value of variables, we convert the continuous variables to the categorical variables. The optimal model predicting OS was verified by the maximal C-index and minimal AIC. OS was defined as the time from the date of surgery to death or last follow-up. The Kaplan-Meier (KM) curves were presented to describe the OS of each subgroup. A nomogram containing LODDS was finally constructed, with the aim to predict the OS of patients. The calibration curves and time-dependent ROC curves within 36 months were exhibited to estimate the nomogram’s predictive capability. DCA is widely applied to evaluate prediction models with the advantage of integrating patients or decision-makers preferences into the analysis and is increasingly used in clinical studies. We presented the DCA curves to analyze the clinical benefits of the nomogram compared with that of the AJCC stage and single LODDS. All statistical analysis in our study was completed by R software (version 4.0.2) and SPSS (version 26.0), the main utilized R packages were “ggplot2”, “Cschange”, “rms” and “timeROC”. For all statistical tests, a *p*-value less than 0.05 in two-sided was regarded as statistically significant.

## Results

### Patients’ Characteristics

According to the CS Schema v0204+, we extracted 15811 pCCA patients meet the inclusion criteria. Finally, we obtained a cohort containing 319 surgical patients between 2010 and 2015 based on the exclusion criteria. All the 319 patients undergoing surgery were included in the training cohort from the SEER database. Most of the patients were diagnosed with cholangiocarcinoma (314[98%]), the male (205[64%]) to female (114[36%]) ratio was 1.80: 1, most of the patients were older than 65 years old (188 [59%]), the white race (245 [77%]). In terms of pathological diagnosis, most of the patients were diagnosed with T2 stage (199 [62%]), 7^th^ N0 stage (173[54%]), 8^th^ N0 stage (174 [55%]), M0 (309 [97%]) and moderately differentiated (163[51%]). The tumor size of most patients was smaller than that of 3cm (186 [58%]). Resected LNs in most patients were more than 4 (217 [68%]). Most patients do not have liver metastasis. Besides, there were 174 patients without positive LNs, instead, 145 patients with one more positive LNs in the training cohort.

A total of 109 pCCA patients undergoing surgery were selected as the external validation cohort from the Chinese PLA General Hospital. The pathological diagnosis was cholangiocarcinoma for all the patients, the male (76[70%]) to female (33[30%]) ratio was 2.30: 1, most of the patients were younger than 65 years old (74 [68%]). In terms of pathological diagnosis, most of the patients were diagnosed with T2 stage (66 [61%]), 8^th^ N0 stage (67 [61%]), M0 stage (109 [100%]) and moderately differentiated (66[61%]). The tumor size of most patients was smaller than that of 3cm (64 [59%]). Resected LNs in most patients were more than 4 (84 [77%]). Then, there were 86 patients without positive LNs, instead of 23 patients with one more positive LNs in the external cohort.

The detailed clinicopathologic characteristics and demographics of the two cohorts were shown in **Table 1**.

**Table 1:** Demographics and characteristics of patients in Training and Validation cohorts.

Characteristics	Training cohort (n=319)	Validation cohort (n=109)	*p* value
**Age (year)**	n (%)	n (%)	<0.001
<65	131(41%)	74(68%)	
≥65	188(59%)	35(32%)	
**Race**			<0.001
White	245(77%)	0	
Black	19(6%)	0	
Others	55(17%)	109(100%)	
**Gender**			0.35
Female	114(36%)	33(30%)	
Male	205(64%)	76(70%)	
**Pathology**			0.335
cholangiocarcinoma	314(98%)	109(100%)	
others	5(2%)	0	
**T stage**			0.003
T1	45(14%)	27(25%)	
T2	199(62%)	66(61%)	
T3	54(17%)	6(6%)	
T4	21(7%)	10(9%)	
**7th N stage**			NA
N0	173(54%)	NA	
N1	140(44%)	NA	
N2	6(2%)	NA	
**8th N stage**			0.075
N0	174(55%)	67(61%)	
N1	111(35%)	38(35%)	
N2	34(11%)	4(4%)	
**M stage**			0.055
M0	309(97%)	109(100%)	
M1	10(3%)	0	
**Grade**			<0.001
Well	57(18%)	3(3%)	
Moderate	163(51%)	66(61%)	
Poor	96(30%)	40(37)	
Undifferentiated	3(1%)	NA	
**Tumor size(cm)**			1
<3	186(58%)	64(59%)	
≥3	133(42%)	45(41%)	
**RLNs**			0.089
<4	102(32%)	25(23%)	
≥4	217(68%)	84(77%)	
**LNR**			0.006
0	244(76%)	68(62%)	
1	75(24%)	41(38%)	
**LODDS**			0.304
1	121(38%)	49(45%)	
2	156(49%)	44(40%)	
3	42(13%)	16(15%)	
**Liver metastasis**			NA
No	313(98%)	NA	
Yes	6(2%)	NA	

LNR lymph node ratio, LODDS the log odds of metastatic lymph nodes, NA not available, RLNs retrieved lymph nodes.

### The Optimal Threshold of LNR and LODDS

To better guide clinical practice, two continuous variables LNR and LODDS were classified *via* the X-tile software which can calculate the optimal threshold among the different groups. In our study, the LNR ranged from 0 to 1, and the LODDS ranged from -2.03 to 1.18. As shown in **Table 2**, the LNR was grouped into LNR1 (LNR<0.27) and LNR2 (LNR≥0.27), and LODDS was classified into LODDS1 -2.03≤LODDS<-0.88, LODDS2 -0.88<LODDS≤-0.16, and LODDS3 LODDS>-0.16. Relevant LNR and LODDS groupings were also shown in **Table 1**.

**Table 2:** The optimal cut-off value of LNR and LODDS.

Variables	Numbers of patients	DR	RR	*p* value
**LNR**				<0.001
LNR<0.27	246	40.24	1	
LNR≥0.27	73	73.97	1.84	
**LODDS**				<0.001
-2.03≤LODDS<-0.88	121	32.23	1	
-0.88<LODDS≤-0.16	156	52.56	1.63	
LODDS>-0.16	42	76.19	2.36	

DR dead rate, RR relative risk.

### Cox Regression Analysis and Identification of Prognostic Factors

**Figure 1:**
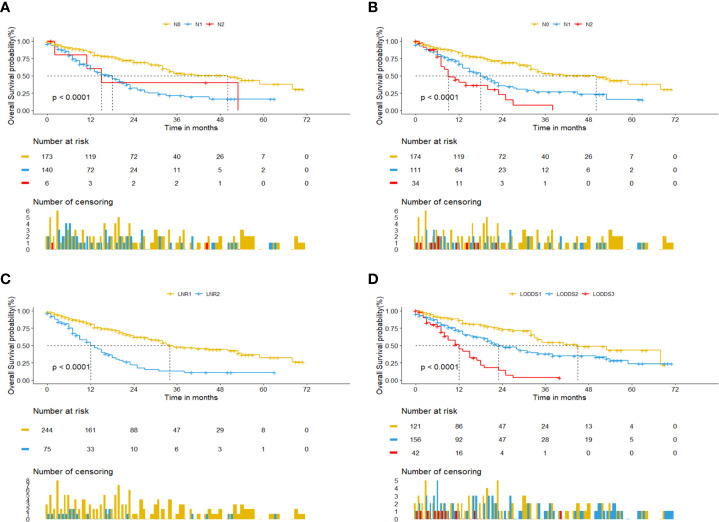
Kaplan-Meier curves for pCCA patients based on four lymph node evaluation factors. **(A)** 7^th^ N stage. **(B)** 8^th^ N stage. **(C)** LNR. **(D)** LODDS.

Univariate cox regression analysis revealed that age of diagnosis (*p*=0.018), race (*p*=0.009), T stage (*p*<0.001), M stage (*p*=0.031), tumor size (*p*=0.007) were potential prognostic factors except four lymph node evaluation factors. The results also revealed the four lymph node evaluation factors (7^th^ N stage, 8^th^ N stage, LNR, and LODDS) also achieved statistical significance(*p*<0.001). Then the age of diagnosis, race, T stage, M stage, and tumor size were included in the first multivariate cox regression analysis. The results revealed that age of diagnosis, T stage, and tumor size could be deemed as independent prognostic factors(*p*<0.05). The detailed results were shown in **Table 3**. Finally, we established four models containing the age of diagnosis, T stage, tumor size and one of four lymph node evaluation factors. As shown in **Table 4**, the second multivariate cox regression analysis showed the 7^th^ N1 stage, 8^th^ N stage, LNR2, and LODDS2,3 were independent prognostic factors(*p*<0.05). The KM curves exhibited that the four lymph node evaluation factors managed to significantly discriminate the OS of each group (**Figures 1A–D**).

**Table 3:** Univariate and multivariate Cox analysis of prognostic factors.

Variables	Univariate analysis			Multivariate analysis		
	HR	95%CI	*p* value	HR	95%CI	*p* value
**Age**	1.49	1.07-2.08	0.018			
<65				Reference		
≥65				1.71	1.20-2.43	0.003
**Race**	1.29	1.07-1.57	0.009			
White				Reference		
Black				1.82	0.95-3.49	0.07
Others				1.42	0.94-2.14	0.098
**T Stage**	1.55	1.29-1.87	<0.001			
T1				Reference		
T2				3.12	1.61-6.02	<0.001
T3				6.02	2.91-12.45	<0.001
T4				4.09	1.78-9.43	<0.001
**M Stage**	2.31	1.08-4.94	0.031			
M0				Reference		
M1				1.82	0.80-4.12	0.151
**Tumor size**	1.54	1.12-2.12	0.007			
<3				Reference		
≥3				1.4	1.00-1.94	0.048
**Gender**	0.96	0.69-1.34	0.816			
**Pathology**	0.41	0.06-2.94	0.376			
**Grade**	1.22	0.95-1.57	0.117			
**RLNs**	0.97	0.7-1.36	0.867			
**7th N Stage**	2.15	1.65-2.82	<0.001			
**8th N Stage**	2.09	1.67-2.62	<0.001			
**LNR**	2.95	2.09-4.12	<0.001			
**LODDS**	2.2	1.71-2.82	<0.001			
**Liver metastasis**	1.56	0.58-4.23	0.379			

**Table 4:** Evaluation of four models.

(a) Multivariate Cox analysis of prognostic factors including 7^th^ N stage			
Variables	Multivariate analysis			
	HR	95%CI	*p* value
**Age**			
<65	Reference		
≥65	1.52	1.084-2.132	0.015
**T stage**			
T1	Reference		
T2	2.73	1.405-5.292	0.003
T3	3.87	1.825-8.217	<0.001
T4	2.64	1.122-6.239	0.026
**Tumor size**			
<3	Reference		
≥3	1.45	1.044-2.014	0.027
**7th N stage**			
N0	Reference		
N1	2.10	1.470-3.007	<0.001
N2	1.46	0.521-4.098	0.471
**C-index**	0.680		
**AIC**	1501.86		
**(b) Multivariate Cox analysis of prognostic factors including 8^th^ N stage**			
**Variables**	**Multivariate analysis**			
****	**HR**	**95%CI**	***p* value**
**Age**			
<65	Reference		
≥65	1.475	1.049-2.047	0.026
**T stage**			
T1	Reference		
T2	2.717	1.400-5.273	0.003
T3	3.716	1.736-7.955	<0.001
T4	2.699	1.145-6.358	0.023
**Tumor size**			
<3	Reference		
≥3	1.401	1.011-1.942	0.043
**8th N Stage**			
N0	Reference		
N1	1.878	1.306-2.703	<0.001
N2	2.709	1.580-4.645	<0.001
**C-index**	0.685		
**AIC**	1497.66		
**(c) Multivariate Cox analysis of prognostic factors including LNR**			
**Variables**	**Multivariate analysis**			
	**HR**	**95%CI**	***p* value**
**Age**			
<65	Reference		
≥65	1.502	1.070-2.109	0.019
**T stage**			
T1	Reference		
T2	2.778	1.434-5.380	0.0024
T3	4.64	2.222-9.687	<0.001
T4	3.16	1.362-7.331	0.0074
**Tumor size**			
<3	Reference		
≥3	1.387	1.001-1.922	0.049
**LNR**			
0	Reference		
1	2.343	1.653-3.321	<0.001
**C-index**	0.685		
**AIC**	1493.9		
**(d) Multivariate Cox analysis of prognostic factors including LODDS**			
**Variables**	**Multivariate analysis**			
****	**HR**	**95%CI**	***p* value**
**Age**			
<65	Reference		
≥65	1.538	1.098-2.154	0.012
**T stage**			
T1	Reference		
T2	2.685	1.384-5.207	0.003
T3	4.569	2.190-9.529	<0.001
T4	3.184	1.371-7.393	0.007
**Tumor size**			
<3	Reference		
≥3	1.297	0.932-1.804	0.122
**LODDS**			
1	Reference		
2	1.636	1.113-2.405	0.012
3	3.572	2.176-5.862	<0.001
**C-index**	0.695		
**AIC**	1491		

AIC Akaike information criterion, C-index concordance index

### Development and Validation of a Nomogram Predicting OS

**Figure 2:**
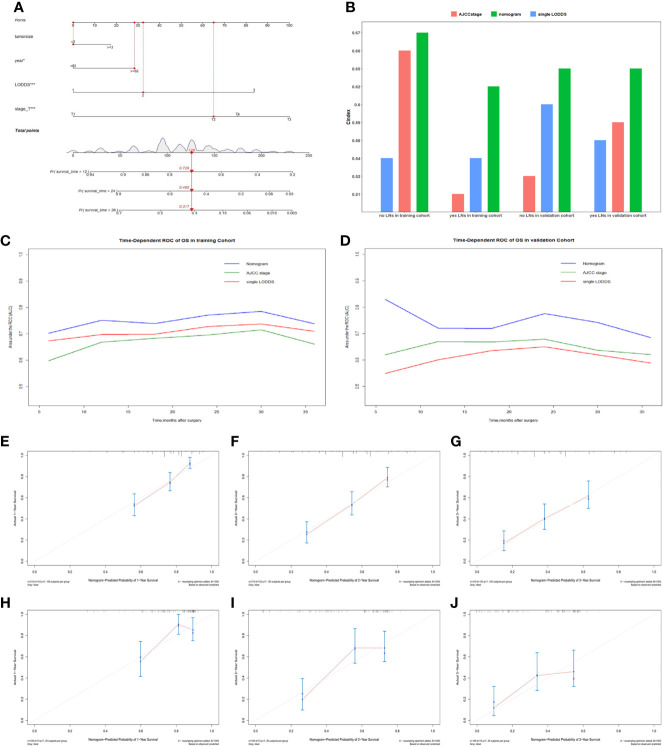
**(A)** The nomogram for predicting OS for pCCA patients. **(B)** Boxplot of C-index in four subgroups. **(C)** The time-dependent AUC of the nomogram in training cohort. **(D)** The time-dependent AUC of the nomogram in the validation cohort. **(E–G)** Calibration curves showed the probability of 1-, 2-, and 3-year OS between the nomogram prediction and the practical observation in the training cohort. **(H–J)** Calibration curves reveal the probability of 1-, 2-, and 3-year OS between the nomogram prediction and the practical observation in the validation cohort.

**Figure 3:**
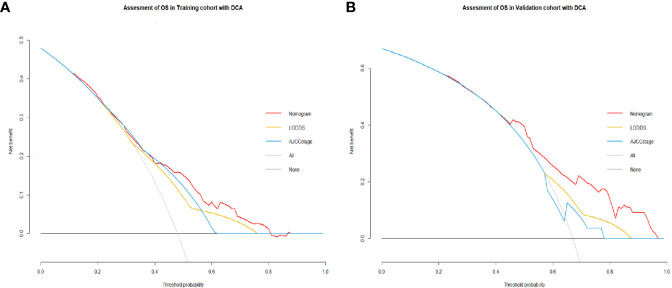
**(A)** Decision curves showed clinical benefits of the nomogram predicting OS in the training cohort. **(B)** Decision curves showed clinical benefits of the nomogram predicting OS in the validation cohort.

Comparisons of discriminability among four models were conducted in these two cohorts. In the training cohort, the C-index of models based on the 7^th^ stage, 8^th^ stage, LNR, LODDS were 0.680, 0.685, 0.685, 0.695, respectively. The AIC was 1501.86, 1497.66, 1493.90, 1491 for the 7^th^ stage, 8^th^ stage, LNR, LODDS (**Table 4**). The maximal C-index and the minimal AIC were distinguished in the model containing LODDS, this result showed this model had the more excellent predictivity for OS, and the LODDS could be regarded as the strongest prognostic factor among the four lymph node evaluation factors. Based on the above results, A prognostic nomogram predicting 1-, 2-, and 3- OS was constructed and an example was exhibited (**Figure 2A**). the C-index of the nomogram was 0.695(95%CI 0.652 to 0.738) that showed it had superior predictive capability compared with the AJCC stage (0.655, 95%CI 0.611 to 0.698, *p* = 0.043) in the training cohort. Similarly, the C-index of the nomogram was 0.688 (95%CI 0.626 to 0.749) that also showed it had superior predictive capability than that of the AJCC stage (0.592, 95%CI 0.525 to 0.660, *p* = 0.019) in the validation cohort. As shown in **Figure 2B**, we further explored the significance of nomogram in the negative LNs group and the positive LNs group, the boxplot showed nomogram with the highest C-index for predicting OS. The time-dependent AUC of the nomogram predicting OS exceeded 0.7 within 36 months in the training cohort (**Figure 2C**), while it exceeded 0.7 within 30months, and was 0.685 at 36 months in the validation cohort (**Figure 2D**). It remains significantly higher than that of AJCC stage and single LODDS, this result showed our nomogram had favorable discrimination. The calibration curves for survival probability showed the nomogram prediction had good uniformity of the practical observation in the training cohort at 1-, 2-, 3-years, similarly, the validation cohort showed similar results (**Figures 2E–J**). Besides, DCA curves exhibited more excellent net benefits in predictive nomogram compared with the AJCC stage and single LODDS among 40%-80% and 45%-95% threshold probability respectively (**Figures 3A, B**). The above results also showed it had wide application in the Eastern and Western.

## Discussion

Perihilar cholangiocarcinoma remains the most common and malignant subtype of biliary tract malignancies (2). Patients with pCCA have a poor prognosis due to late diagnosis and high malignancy (20). Under this condition, lymph node metastasis plays an indispensable prognostic role in tumor progression. Because of increasing importance in assessing patients’ prognosis, various stage criteria (N stage, LNR and LODDS) have been studied by a partial clinician (21). Therefore, we were fully interested in methods of evaluating lymph node metastasis. By univariate and multivariate cox regression, we constructed four models containing independent factors and lymph node evaluation factors. According to the maximal C-index and minimal AIC, an optimal model containing LODDS was selected. Finally, we constructed a nomogram based on this optimal model to predict the OS of pCCA patients, and which had higher clinical benefits than the 8^th^ AJCC stage. The calibration curves showed there were good discriminative and calibration capabilities in this nomogram. To our knowledge, our study firstly reports a nomogram based on LODDS for predicting OS of pCCA patients and performs validation by the population from eastern and western surgery centers.

Previous studies had proposed various significantly prognostic factors for patients with pCCA, such as the age of diagnosis, tumor size, and lymph node metastasis (7, 8). In our study, these key prognostic factors were fully considered. Lurje G et al. and Aoba T et al. study both reported lymph node metastasis was a strong prognostic factor and relevant with shorter OS of patients (13, 22). In the clinical practices, there are various evaluation methods of lymph node metastasis. In the 7^th^ AJCC stage, the N stage was used to evaluate lymph node metastasis by exploring whether there is lymph node metastasis in certain areas (9), but the various clinical studies had proposed that Judging by the metastatic area of LNs had few limitations (8, 21). Consequently, the 8^th^ AJCC stage was newly revised. The number of regional positive LNs was used as the evaluation standard: N0 stage was no positive LNs detected, N1 stage was 1-3 LNs detected, and N2 stage was the more than 4 regionally positive LNs were retrieved (10). However, recently studies indicated it had similar predictive capability compared with the 7^th^ AJCC stage according to the above criteria (11, 12, 23). In our study, according the C-index of models, it was confirmed that the models involving 7^th^ and 8^th^ N stage has comparable predictive accuracy(0.680 vs. 0.685). The reason may be the difference in surgical methods and operative habits of different medical centers, the detected number of LNs was non-uniform, and there was no guarantee that a certain number of LNs can be detected for analysis. In our study, the median retrieved LNs were 6 both in these two cohorts. Although there is no specific requirement for the retrieved number of LNs in the 8^th^ AJCC stage, the N stage lacked the well predictive capability. It revealed that the clinical efficacy of the N stage remains to be further considered and whether extended lymph node dissection is indispensable during operation. Aim to the above problems, LODDS and LNR were discovered by clinicians and hopeful to be used in the clinical practice (24, 25). They contained more information than the N stage, so it is more reasonable than the N stage. Guglielmi A et al. study suggested that LNR has greater predictive power compared to the N stage (26). However, LNR similarly depended on the retrieved number of LNs, it inevitably divided some patients into high LNR group due to the insufficiently retrieved number of LNs leading the underestimated number of positive LNs. Therefore, LODDS, a new method for evaluating lymph node metastasis, was proposed. The calculation of LODDS uses empirical transformation methods. With these statistical characteristics, LODDS has better accuracy to predict patient prognosis. Previous studies have shown that LODDS has well predictive accuracy in various cancers, such as gastric cancer, pancreatic cancer and so on (15, 17). Although some articles have studied the predictive effect of LODDS for pCCA, the predictive capability of it remains to be further verified by an external cohort of mature medical centers.

In this study, by comparing with the other three models by C-index, AIC and other verification indicators, the model containing LODDS is established, with the best predictive capability. Finally, we obtained an optimal model containing four independent prognostic factors: age, T stage, tumor size, and LODDS, and a nomogram was constructed based on the above model. This study also proved that our model had greater predictive value compared with the traditional AJCC stage in the eastern and western cohorts. In addition, the relevant information included in the model was relatively easy to obtain and further evaluated in clinical practice, which would be more convenient for clinical application. We further explored the significance of nomogram in the negative LNs group and the positive LNs group according to the C-index and found that it was better used in these two groups than the AJCC stage and single LODDS in the training cohort, and similarly, better than that in the validation cohort. Besides, the DCA curves demonstrated that our nomogram predicting OS had higher clinical benefits than the traditional AJCC stage and single indicator in these two cohorts. Interestingly, for the population in the validation cohort from China, nomogram had better improvement, the reason may be the different baseline and increasingly mature technique in our surgical center. It could also better reflect the advantages of our nomogram in advanced patients and the prospect of popularization and application in the Eastern and Western, and with the further development of biliary surgery, the application of nomogram may be more extensive.

Although this nomogram had a great performance for predicting the OS of pCCA patients, some limitations also existed in this study. Firstly, the data of the training cohort come from the SEER database, while the validation cohort comes from China, some biases were inevitable because of regional difference, but the results better illustrated the universality of the nomogram application. Besides, our study was a retrospective study with its inherent defects. Secondly, the cut-off value of LODDS in our study is based on X-tile software, although it is significantly suitable for this study population, whether it is suitable for the general population deserved further confirmation from multi-center, large-volume clinical studies. Furthermore, we also need a uniform standard for LODDS stratified truncation value to apply it to clinical work gradually.

## Conclusion

The nomogram had importantly potential value in clinical practice. Compared with the AJCC stage, our nomogram could be regarded as a more accurate and credible method predicting the OS of pCCA patients, which has more clinical utility and more convenience to guide clinical practice. Our study also revealed LODDS is an accurately prognostic factor than other N stage and LNR. Hopefully, LODDS could promote the development of the novel stage for pCCA and guide the implementation of personalized treatment.

## Data Availability Statement

The raw data supporting the conclusions of this article will be made available by the authors, without undue reservation.

## Author Contributions

WZ, CZ, and ZW contributed equally to this work. WZ and CZ: data collection and manuscript writing. ZW: data processing and interpretation. XT, CL, ZZ, and MH: data acquisition. RL: problem posing and critical revision. All authors contributed to the article and approved the submitted version.

## Conflict of Interest

The authors declare that the research was conducted in the absence of any commercial or financial relationships that could be construed as a potential conflict of interest.
